# Experiência de um Centro Brasileiro com Crioablação para Isolamento Elétrico das Veias Pulmonares na Fibrilação Atrial Paroxística e Persistente – Resultados Preliminares no Brasil

**DOI:** 10.36660/abc.20190307

**Published:** 2020-09-18

**Authors:** Silvia Helena Cardoso Boghossian, Eduardo C. Barbosa, Eduardo Boghossian, Lucas Rangel, Paulo Roberto Benchimol-Barbosa, Mônica Luiza de Alcantara, Marcio Fagundes, Alex Felix, Ricardo Mourilhe-Rocha

**Affiliations:** 1 Universidade do Estado do Rio de Janeiro Rio de Janeiro RJ Brasil Universidade do Estado do Rio de Janeiro - UERJ, Rio de Janeiro, RJ - Brasil; 2 Hospital Vitória Hospital Samaritano Barra Rio de Janeiro RJ Brasil Hospital Vitória e Hospital Samaritano Barra, Rio de Janeiro, RJ - Brasil; 3 Hospital Pró-Cardíaco Rio de Janeiro RJ Brasil Hospital Pró-Cardíaco, Rio de Janeiro, RJ - Brasil

**Keywords:** Fibrilação Atrial, Crioablação, Congelamento, Veias Pulmonares

## Abstract

**Fundamento:**

O isolamento elétrico das veias pulmonares é reconhecidamente base fundamental para o tratamento não farmacológico da fibrilação atrial (FA) e, portanto, tem sido recomendado como passo inicial na ablação de FA em todas as diretrizes. A técnica com balão de crioenergia, embora amplamente utilizada na América do Norte e Europa, ainda se encontra em fase inicial em muitos países em desenvolvimento, como o Brasil.

**Objetivo:**

Avaliar o sucesso e a segurança da técnica de crioablação em nosso serviço, em pacientes com FA paroxística e persistente.

**Métodos:**

Cento e oito pacientes consecutivos com FA sintomática e refratária ao tratamento farmacológico foram submetidos à crioablação para isolamento das veias pulmonares. Os pacientes foram separados em dois grupos, de acordo com a classificação convencional da FA paroxística (duração de até sete dias) e persistente (FA por mais de sete dias). Dados de recorrência e segurança do procedimento foram analisados respectivamente como desfechos primário e secundário. O nível de significância adotado foi de 5%.

**Resultados:**

Cento e oito pacientes, com idade média de 58±13 anos, 84 do sexo masculino (77,8%), foram submetidos ao procedimento de crioablação de FA. Sessenta e cinco pacientes apresentavam FA paroxística (60,2%) e 43, FA persistente (39,2%). O tempo médio do procedimento foi de 96,5±29,3 minutos e o tempo médio de fluoroscopia foi de 29,6±11,1 minutos. Foram observadas cinco (4,6%) complicações, nenhuma fatal. Considerando a evolução após os 3 meses iniciais, foram observadas 21 recorrências (19,4%) em período de um ano de seguimento. As taxas de sobrevivência livre de recorrência nos grupos paroxístico e persistente foram de 89,2% e 67,4%, respectivamente.

**Conclusão:**

A crioablação para isolamento elétrico das veias pulmonares é um método seguro e eficaz para tratamento da FA. Nossos resultados estão consoantes com demais estudos, que sugerem que a tecnologia pode ser utilizada como abordagem inicial, mesmo nos casos de FA persistente. (Arq Bras Cardiol. 2020; 115(3):528-535)

## Introdução

O isolamento elétrico das veias pulmonares (IEVP) é considerado fundamento básico para o tratamento ablativo da fibrilação atrial (FA). Estudos relatam uma taxa de sucesso ao redor de 80%, em seguimento de longo prazo, de pacientes com FA paroxística submetidos a esse procedimento.^1^

Nas atuais diretrizes de conduta, brasileiras e internacionais, o IEVP é a estratégia recomendada para ablação de FA paroxística sintomática e refratária a tratamento farmacológico.^2,3^ Inicialmente, essa estratégia era utilizada apenas nos casos de FA paroxística. Porém, estudos recentes envolvendo pacientes portadores de FA persistente compararam o IEVP isoladamente ao IEVP associado a técnicas mais complexas e demonstraram eficácia semelhante.^4-8^ Assim, o isolamento elétrico das veias pulmonares tem sido considerado, atualmente, o passo inicial da ablação mesmo nos casos de FA de mais longa duração.^9^

Além disso, estudos que avaliaram a técnica de isolamento por meio da crioablação com balão tiveram resultados semelhantes aos obtidos com o uso da energia de radiofrequência. Pela sua eficácia, segurança, além de superioridade em relação ao número de reintervenções e hospitalizações, a crioenergia tem sido bastante utilizada na atualidade.^3,10,11^

Tondo et al.,^12^ num estudo multicêntrico, de mundo real, sobre a utilização do balão de crioenergia em pacientes com FA persistente e persistente de longa duração, concluíram que a segurança e eficácia do método são semelhantes ao IEVP por meio da radiofrequência.

## Objetivos

Os objetivos desse estudo foram avaliar a eficácia e a segurança da técnica de crioablação para tratamento da FA na experiência inicial de um centro brasileiro.

## Métodos

Foram realizados 108 procedimentos consecutivos no período de dezembro de 2015 a abril de 2018. Todos os pacientes assinaram o termo de consentimento informado. Em todos os pacientes, o procedimento foi realizado com a segunda geração do balão de crioenergia (Arctic Front Advance, Cardiac Cryoablation Catheter System; Medtronic, Inc Minneapolis, MN).

A FA foi classificada como paroxística se sua duração fosse menor que 7 dias, independentemente de reversão espontânea, química ou elétrica, e persistente se a arritmia permanecesse por mais de 7 dias. Os critérios de exclusão contemplaram pacientes com doença cardíaca estrutural (insuficiência cardíaca congestiva, cardiomiopatia hipertrófica, valvopatia) e átrio esquerdo (AE) maior que 5,5 cm.

Os pacientes em uso de anticoagulante oral de ação direta foram orientados a suspender uma dose da medicação previamente ao procedimento.

**Figura 1:**
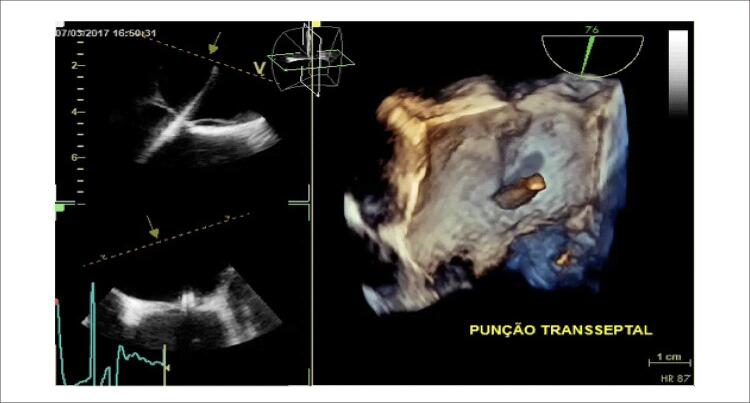
– Punção transeptal guiada pelo ecocardiograma transesofágico 3D.

A crioablação foi realizada sob anestesia geral e após infusão de bolus de 5000 UI de Heparina. A punção transeptal para acesso ao átrio esquerdo foi guiada pelo ecocardiograma transesofágico (Figura 1). Mais 5000 UI de heparina foram infundidas após o acesso ao mesmo. Os pacientes que chegaram à sala de eletrofisiologia em vigência de FA foram submetidos à cardioversão elétrica antes do procedimento.

**Figura 2:**
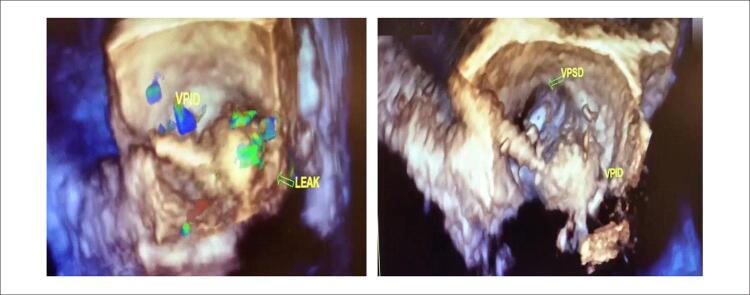
– Posicionamento do balão guiado pelo ecocardiograma transesofágico 3D. À esquerda: oclusão insatisfatória. Observa-se o vazamento do contraste em torno do balão. À direita: oclusão satisfatória. Não se observa vazamento do contraste.

**Figura 3:**
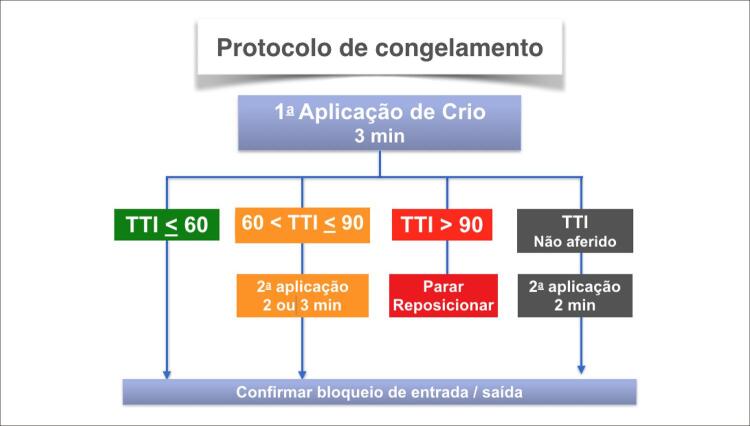
– Protocolo de liberação da crioenergia. TTI: tempo total para isolamento elétrico da veia.

O balão de crioablação de 28 mm e o cateter guia de mapeamento circular (Achieve) foram introduzidos no AE através da bainha flexível específica para o sistema (FlexCath, Medtronic, Inc.). O posicionamento do balão e a oclusão das VP foram confirmados por fluoroscopia e pelo ecocardiograma 3D (Figura 2). O número de aplicações de crioenergia e a duração do procedimento variaram em função do tempo para atingir o isolamento elétrico em cada veia; se o isolamento fosse observado em até 60 segundos, era feita apenas uma aplicação de 180 segundos. Se o isolamento fosse observado entre 60 e 90 segundos, era realizada uma segunda aplicação de 120 segundos. Quando o tempo de isolamento não podia ser mensurado devido à necessidade de se avançar o cateter para melhor posicionamento e oclusão do balão, eram liberadas duas aplicações de 180 segundos (Figura 3). A temperatura mínima admitida para as veias esquerdas foi de -60 ^o^C e, para as veias direitas, de -55 ^o^C. Se esses valores fossem ultrapassados, a aplicação de crioenergia era interrompida. Após o congelamento, a eficácia do isolamento elétrico das veias foi confirmada pelo bloqueio bidirecional nas mesmas.

O seguimento dos pacientes foi realizado por meio de visitas médicas e Holter de 24 horas após 30, 60 e 90 dias, seis, nove e 12 meses. Em caso de relato de sintomas, foi indicado Holter prolongado ou monitor de eventos externo.

Nos primeiros 3 meses de seguimento (*blanking period*), as drogas antiarrítmicas (DAA) foram mantidas em todos os pacientes. Após esse período, foram suspensas nos portadores de FA paroxística. Nos pacientes com FA persistente prévia ao procedimento, a decisão sobre suspensão ou não das drogas foi individualizada e variava em função de diversos fatores, como o tempo de evolução da FA, o tamanho do AE e a presença de comorbidades.

Definiu-se recidiva como o registro eletrocardiográfico de FA com mais de 30 segundos de duração, independentemente do uso de DAA.

### Análise estatística

Variáveis contínuas foram expressas pela média e desvio padrão e analisadas peto teste *t* de Student não pareado após constatação de distribuição normal pelo teste Shapiro-Wilk. Variáveis categóricas foram expressas em porcentagem e analisadas pelo teste X^2^. Taxas livres de eventos de fibrilação atrial foram calculadas pelo método de Kaplan-Meier e analisadas utilizando-se o modelo preditivo de risco proporcional de Cox. Foram utilizados os aplicativos MedCalc versão 10.3.2 (MedCalc software bvba, Ostend, Belgium; https://www.medcalc.org; 2016) e MS-Excel 2010 (Microsoft Corporation). O nível de significância estatística foi estabelecido em 5%.O nível de significância estatística foi estabelecido em 5%.

## Resultados

Dos 108 pacientes submetidos ao procedimento de crioablação, 65 (60,2%) eram portadores de FA paroxística e 43 (39,2%), de FA persistente. A idade média foi de 58±13 anos (entre 28 e 84 anos) e 84 pacientes eram do sexo masculino (77,8%). O tempo médio do procedimento, medido a partir da punção transeptal (tempo de AE) até o isolamento das quatro veias pulmonares foi de 96,5±29,3 minutos, e o tempo médio de fluoroscopia, de 29,5±11,1 minutos. O tempo médio de seguimento foi de 367±20 dias.

**Figura 4:**
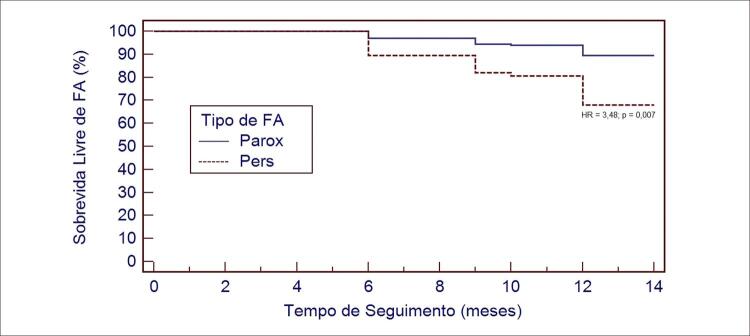
– Sobrevida livre de recorrência de fibrilação atrial em um ano pela curva de Kaplan Meyer.

Finalizados os três primeiros meses após a ablação (*blanking period*), 21 (19,4%) pacientes apresentaram recidiva da FA. O grupo de pacientes com FA paroxística apresentou menor taxa de recorrência em relação ao grupo com FA persistente, respectivamente sete (10,8%) e 14 (32,5%) pacientes: p=0,007, HR: 3,48 (1,41 a 8,59). (Figura 4).

O grupo com FA persistente tinha idade mais avançada, maior número pacientes com átrio esquerdo dilatado e escore de CHA_2_DS_2_VASc≥3 (Tabela 1). Contudo, essas variáveis não foram preditivas do desfecho primário na análise univariada (Tabela 2).

**Tabela 1:** Variáveis demográficas e clínicas

	FA paroxística	FA persistente	p
N	65	43	
Idade (anos)	55,1±13,1	62,8±10,8	0,002
Sexo masculino	80,0%	74,4%	0,87
AE aumentado	20,0%	69,7%	<0,001
CHA_2_DS_2_VASc≥3	20,0%	37,0%	0,08

*FA: fibrilação atrial; AE: átrio esquerdo.*

**Tabela 2:** – Variáveis e respectivas razões de risco para recorrência de FA em até 1 ano (Modelo proporcional de Cox Univariado)

	HR	IC 95%	p
Idade (anos)	1,02	[0,99–1,06]	0,10
Sexo masculino	1,04	[0,35–3,06]	0,95
AE aumentado	1,94	[0,84–4,48]	0,08
CHA_2_DS_2_VASc≥3	1,80	[0,77–4,19]	0,13
Tipo de FA	3,48	[1,41–8,59]	0,007
Recorrência BP	3,37	[1,41–8,12]	0,007

*AE: átrio esquerdo; FA: fibrilação atrial; BP: blanking period.*

**Figura 5:**
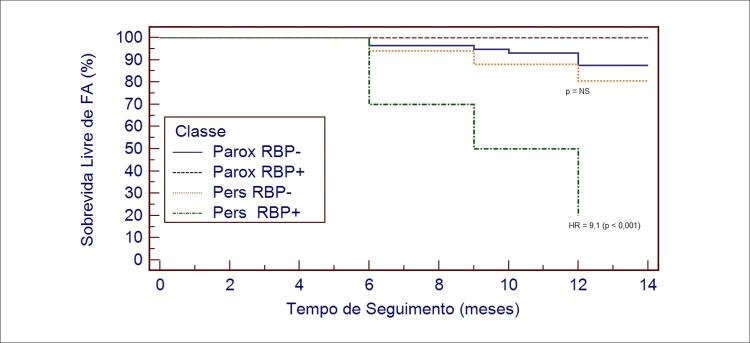
– Sobrevida livre de fibrilação atrial comparando pacientes com ou sem recorrência no blanking period pela curva de Kaplan Meyer. Parox: FA paroxística. Pers: FA persistente. RBP: recorrência no blanking period.

A recorrência no *blanking period* foi observada em 18 pacientes (16,7%) e foi fator preditivo de recorrência tardia no grupo de pacientes com FA persistente (Figura 5).

Entre os oito casos com recorrência precoce no grupo com FA paroxística, nenhum apresentou recorrência durante o seguimento clínico tardio, enquanto nos 10 casos do grupo com FA persistente, oito apresentaram recorrência tardia.

Foram observadas complicações menores em cinco (4,6%) pacientes: um caso de derrame pericárdico com resolução espontânea; 2 casos de paralisia autolimitada do nervo frênico (PNF), recuperada espontaneamente em 15 minutos, um caso de PNF persistente após a alta hospitalar e um caso de pseudoaneurisma de artéria femoral, tratado e resolvido clinicamente. Não foram observados sangramentos maiores, acidentes encefálicos ou morte durante ou após o procedimento.

## Discussão

A FA é a arritmia sustentada mais comum na população em geral e, independentemente do tipo de energia ou da técnica utilizada, o isolamento completo das veias pulmonares é a estratégia mais eficaz, tendo se tornado o principal objetivo do tratamento ablativo da FA.^2-10^ Inicialmente, essa técnica era indicada apenas na FA paroxística, até que estudos subsequentes demonstraram sua não inferioridade em relação a outros procedimentos mais complexos e abrangentes, especialmente em pacientes com FA persistente.^4-9^

O Estudo Fire and Ice foi o primeiro grande estudo multicêntrico randomizado que comparou os resultados do uso do criobalão e da energia de radiofrequência na ablação da FA paroxística e demonstrou sua não inferioridade, tanto em relação à eficácia quanto à segurança do procedimento.^10,11^ A análise dos objetivos secundários do estudo demonstrou benefícios do balão quando consideradas as taxas de internação hospitalar, necessidade de cardioversão e reintervenção.^11^ Esses benefícios foram confirmados por Mörtsell et al.,^13^ que publicaram recentemente os resultados de eficácia e segurança do procedimento baseado nos registros de ablação do ESC-EHRA e no registro sueco.

A FA persistente apresenta um substrato mais complexo e o índice de sucesso com o IEVP é mais limitado.^2,14^ Com o objetivo de reduzir o índice de recorrência, estratégias mais abrangentes foram adotadas, como a realização de linhas de bloqueio adicionais e a ablação de eletrogramas atriais fracionados.^2^ No entanto, o benefício adicional dessas ablações mais extensas, de acordo com estudos comparativos recentes, permanece controverso.^6,9^ Assim sendo, de acordo com as diretrizes internacionais, o IEVP ainda é o alvo final do procedimento de ablação da FA e técnicas que abrangem áreas de ablação mais extensas não têm sido recomendadas numa primeira intervenção.^2^

Embora a ablação com radiofrequência seja considerada o padrão ouro para a FA persistente, estudos com o balão de crioenergia têm demonstrado resultados clínicos satisfatórios.^13,15^

O estudo CRYO4PERSISTENT, publicado recentemente, avaliou não apenas a recorrência de FA, mas, também que a presença de sintomas pós IEVP com o balão de crioenergia demonstrou melhora significativa da qualidade de vida dos pacientes.^15^ Esses achados também foram confirmados no estudo de Mörtsell et al.^13^ que relatou menor recorrência de sintomas e menor uso de drogas antiarrítmicas no grupo que realizou a ablação com o balão.

Em nosso estudo, descrevemos a primeira experiência de um centro brasileiro que realizou o IEVP utilizando o balão de crioenergia como abordagem inicial para o tratamento não farmacológico da fibrilação atrial em um número grande de pacientes. No seguimento de um ano, a taxa livre de eventos foi de 89,2% para o grupo com FA paroxística e de 67,4% naquele com FA persistente. No CIRCA-DOSE trial, recentemente apresentado, no qual a avaliação da recidiva foi feita por meio de monitor de eventos eletrocardiográficos implantável, a taxa livre de recorrência foi em torno de 64%. No entanto, a taxa livre de FA sintomática foi próxima a 80%.^11-15^ Em nosso estudo, o tempo médio de procedimento no AE foi de 96,5±29,3 minutos e o tempo de fluoroscopia, de 29,5±11,1 minutos, duração próxima às reportadas nos diversos estudos realizados.^12,13^

Em relação à segurança, observamos um índice de complicações de 4,6%, o que é considerado bastante satisfatório e similar aos relatados na literatura.^10,12^A complicação mais frequentemente observada foi a paralisia de nervo frênico, que ocorreu em 3 pacientes. Em dois casos, a paralisia foi transitória, e reverteu-se ainda na sala de eletrofisiologia. Em uma paciente, a paralisia foi persistente, e a paciente foi encaminhada para fisioterapia. Houve apenas um caso de complicação vascular. Consideramos que o baixo índice dessa complicação se deva ao fato de todas as punções terem sido guiadas por ultrassom.

Um fato importante a ser discutido é que o principal mecanismo destrutivo da crioablação é a lise celular causada pela formação de gelo no meio intra e extra celular, causando um desequilíbrio osmótico. Esse desequilíbrio provoca rotura da membrana celular e danos nas estruturas celulares, acarretando morte celular por necrose e por apoptose. Assim, observou-se menor resposta inflamatória na ablação e, consequentemente, menor edema.^16^ A inflamação é um dos fatores apontados como responsáveis pela reconexão das veias. Outro fato importante a ser mencionado na ablação por criotermia é que esta não promove desnaturação de proteínas, preservando o colágeno e a elastina do tecido conjuntivo e, consequentemente, preservando a matriz extracelular. Assim, há redução do risco de formação de trombos, de estenose das veias e de lesão no esôfago.^17^ Em nossa casuística, não houve nenhum caso de fístula esofágica, evidência clínica ou laboratorial de estenose de veias pulmonares ou morte.

Nossa experiência inicial é semelhante à dos demais estudos publicados na literatura^11-16^ e confirma que os resultados obtidos com a crioablação são reprodutíveis e menos operador-dependentes do que aqueles das ablações por radiofrequência,^11-16^necessitando, portanto, de uma curva de aprendizado menos prolongada.

## Limitações

A principal limitação deste trabalho é se tratar de um estudo observacional realizado em centro único, sem grupo controle e, portanto, pode ter ocorrido um viés na seleção de pacientes, com menos comorbidades, já que foram encaminhados por médicos atuantes em clínicas privadas.

Além disso, é um seguimento de curto prazo de uma tecnologia que está sendo introduzida em nosso país e, portanto, não está disponível para uso em grande escala, o que dificultou a inclusão de maior número de pacientes.

Estudos randomizados de outros centros especializados contendo maior número pacientes e com seguimento clínico mais prolongado devem ser realizados para confirmar nossos resultados.

## Conclusão

A crioablação para isolamento elétrico das veias pulmonares revelou-se um método seguro e eficaz, com baixos níveis de complicação e resultados bastante satisfatórios. Nossos resultados estão de acordo com os demais estudos da literatura, que sugerem que essa tecnologia pode ser utilizada como abordagem inicial não farmacológica para o tratamento da FA, mesmo nos casos de FA persistente.
