# Errata

**DOI:** 10.36660/abc.20200807

**Published:** 2020-08-19

No Artigo Original “Experience in a Brazilian Center with Crioablation for Electric Isolation of the Pulmonary Veins in Paroxysmal and Persistent Atrial Fibrillation – Preliminary Results in Brazil”, com número de DOI: : https://doi.org/10.36660/abc.20200320, publicado no periódico Arquivos Brasileiros de Cardiologia, corrigir, no título em inglês, a palavra Crioablation para Cryoablation, corrigir o nome do autor Eduardo Barbosa para Eduardo C. Barbosa e alterar o número de DOI para DOI: https://doi.org/10.36660/abc.20190307.

