# Supplementary material for: Experiência de um Centro Brasileiro com Crioablação para Isolamento Elétrico das Veias Pulmonares na Fibrilação Atrial Paroxística e Persistente – Resultados Preliminares no Brasil
Source: Arq Bras Cardiol. 2020 Sep 18;115(3):528–35. [Article in Portuguese] doi: 10.36660/abc.20190307 (PMC9363100; doi:10.36660/abc.20190307)
Supplement: Translation

# Translation: Experience in a Brazilian Center with Cryoablation for Electric Isolation of the Pulmonary Veins in Paroxysmal and Persistent Atrial Fibrillation – Preliminary Results in Brazil

**Authors:** Silvia Helena Cardoso Boghossian, Eduardo C. Barbosa, Eduardo Boghossian, Lucas Rangel, Paulo Roberto Benchimol-Barbosa, Mônica Luiza de Alcantara, Marcio Fagundes, Alex Felix, Ricardo Mourilhe-Rocha

**Affiliations:** 1 Brazil Universidade do Estado do Rio de Janeiro - UERJ, Rio de Janeiro, RJ - Brazil; 2 Brazil Hospital Vitória e Hospital Samaritano Barra, Rio de Janeiro, RJ - Brazil; 3 Brazil Hospital Pró-Cardíaco, Rio de Janeiro, RJ - Brazil

**Keywords:** Atrial Fibrillation, Cryoablation, Freezing, Pulmonary Veins

## Abstract

**Background:**

Electrical isolation of the pulmonary veins is recognized as the cornerstone of non-pharmacological treatment of Atrial Fibrillation (AF), and therefore, has been recommended as the first step in AF ablation according to all guidelines. Even though the cryoballoon technology is widely used in North America and Europe, this experience is still incipient in many developing countries such as Brazil.

**Objective:**

To evaluate initial results regarding success and safety of the new technology in patients with persistent and paroxysmal AF.

**Methods:**

One hundred and eight consecutive patients with symptomatic AF refractory to pharmacological treatment were submitted to cryoablation for isolation of the pulmonary veins. Patients were separated into two groups according to AF classification: persistent (AF for over one week); or paroxysmal (shorter episodes). Recurrence and procedural safety data were analyzed respectively as primary and secondary outcomes. The level of significance was 5%.

**Results:**

One hundred and eight patients, with mean age 58±13 years, 84 males (77.8%), underwent cryoablation. Sixty-five patients had paroxysmal AF (60.2%) and 43 had persistent AF (39.2%). The mean time of the procedure was 96.5±29.3 minutes and the mean fluoroscopy time was 29.6±11.1 minutes. Five (4.6%) complications were observed, none fatal. Considering a blanking period of 3 months, 21 recurrences (19.4%) were observed in a one-year follow-up period. The recurrence-free survival rates of AF in the paroxysmal and persistent groups were 89.2% and 67.4%, respectively.

**Conclusion:**

Cryoablation for electrical isolation of the pulmonary veins is a safe and effective method for the treatment of AF. Our results are consistent with other studies suggesting that this technology can be used as an initial technique even in cases of persistent AF. (Arq Bras Cardiol. 2020; 115(3):528-535)

## Introduction

Electrical isolation of the pulmonary veins (EIPV) is considered the cornerstone of the treatment of atrial fibrillation (AF). Studies report more than 80% success in long-term follow-up in patients with paroxysmal AF.^1^

In the current guidelines, EIPV is the recommended strategy for non-pharmacological treatment of atrial fibrillation in patients with paroxysmal AF, symptomatic and refractory to pharmacological treatment.^2,3^ This strategy was initially used only in cases of paroxysmal AF, but consistent results in several studies demonstrated that it presented results similar to other more complex approaches in cases of persistent AF.^4-8^ EIPV is currently the index procedure in cases of persistent AF.^9^

Studies using balloon cryoablation with EIPV have shown similar results to radiofrequency energy use in relation to efficacy and safety and some superiority regarding the number of re-interventions and hospitalizations.^3,10,11^

Tondo et al.^12^ have recently published a multicenter study with real-world results on the use of the cryoenergy balloon in patients with persistent and long-standing persistent AF and concluded that safety and efficacy of the method are similar to those of EIPV through radiofrequency.

### Objective

The main objective of our study was to evaluate the initial results of the use of cryoablation for initial treatment of AF in our setting regarding the efficacy and safety of the procedure.

## Methods

One hundred and eight procedures were performed, from December 2015 to April 2018. All patients signed a written informed consent form. In all patients, the procedure was performed with the second-generation cryoenergy balloon (Arctic Front Advance Cardiac Cryoablation Catheter System, Medtronic, Inc Minneapolis, MN).

AF was classified as paroxysmal if the duration of the episodes was shorter than 7 days, even if chemically or electrically cardioverted; and persistent if there were any episodes lasting longer than 7 days. Patients with significant structural heart disease (congestive heart failure, hypertrophic cardiomyopathy, valvular heart disease) or left atrium (AE) greater than 5.5 cm were excluded from this study.

Patients on direct-acting oral anticoagulant were instructed to suspend one dose of the medication prior to the procedure.

Cryoablation was performed under general anesthesia. Heparin bolus 5000 IU was administered prior to transesophageal echocardiogram-guided transseptal needle puncture for access to the left atrium (Figure 1), followed by an additional 5000 IU heparin after transseptal puncture. Patients who arrived at the electrophysiology room in AF were submitted to electrical cardioversion before the procedure.

**Figure 1:** – Transseptal puncture guided by 3D transesophageal echocardiography.

The 28 mm cryoablation balloon and the circular mapping catheter (Achieve) were introduced into the LA through system-specific flexible cuff (FlexCath, Medtronic, Inc.). The ideal balloon positioning and PV occlusion were confirmed by fluoroscopy and by 3D echocardiogram (Figure 2). The number and time of cryoenergy applications in each vein varied as a function of the time necessary to achieve electrical isolation of the vein: if insulation was observed within 60 seconds, only one application of 180 seconds was made; if insulation was observed between 60 and 90 seconds, a second 120-second freezing cycle was applied; when the insulation time could not be measured due to the need to advance the catheter for better balloon positioning and PV occlusion, two applications of 180 seconds were used (Figure 3). The minimum allowed temperature for the left veins was -60 °C and, for the right veins, -55 °C; if these temperatures were exceeded, energy supply would be immediately interrupted. After freezing, the effectiveness of the electrical insulation of the veins was confirmed by bidirectional blocking of electrical stimuli through them.

**Figure 2:** – Balloon positioning guided by 3D transesophageal echocardiography. Left: unsatisfactory occlusion. Contrast leakage around the balloon. Right: satisfactory occlusion. No contrast leakage.

**Figure 3:** – Freezing Protocol. TTI: time to isolation.

Patient follow-up was performed with medical visits and 24-hour Holter at 30, 60 and 90 days, 6, 9, 12 months. If any symptoms were reported between the visits, graphic documentation of potential arrhythmias was instituted with prolonged Holter or event monitor. In the first 3 months of follow-up (blanking period) antiarrhythmic drugs (AADs) were maintained on all patients. After this period, it was discontinued for all paroxysmal AF patients. In persistent AF patients, the decision to suspend the drugs was individualized and varied due to several factors such as the time of AF evolution, LA size and the presence of comorbidities. Recurrence was defined as graphic documentation of AF lasting over 30 seconds, regardless of the use of AADs.

### Statistical analysis

Continuous variables were expressed as mean and standard deviation and analyzed by unpaired Student’s t-test after finding a normal distribution by the Shapiro-Wilk test. Categorical variables were expressed as percentage and analyzed by X^2^. Atrial fibrillation events were calculated using the Kaplan-Meier method and analyzed using the Cox proportional risk predictive model. MedCalc version 10.3.2 (MedCalc software bvba, Ostend, Belgium; https://www.medcalc.org; 2016) and MS-Excel 2010 (Microsoft Corporation) applications were used. The level of statistical significance was set at 5%.

## Results

Among 108 patients with atrial fibrillation submitted to cryoablation for electrical isolation of the pulmonary veins, 65 (60.2%) had paroxysmal AF and 43 (39.2%) had persistent AF. Mean age was 58±13 years (28-84) and 84 patients were males (77.8%). The mean time of the procedure, measured from the transseptal puncture, (LA time) was 96.5±29.3 minutes and the mean time of fluoroscopy was 29.5±11.1 minutes. The mean follow-up time was 367±20 days.

After the blanking period (3 months after the procedure), 21 patients had relapsed AF (19.4%). The paroxysmal group had a lower recurrence rate of AF than the persistent one: 7 (10.8%) for the paroxysmal group and 14 (32.5%) for the persistent group: p=0.007, HR: 3.48 (1.41 to 8.59) (Figure 4).

**Figura 4:** – One year atrial fibrillation-free survival by Kaplan-Meyer curve.

The persistent AF group presented, compared to the paroxysmal, higher age and higher number of cases with left atrial enlargement and CHA2DS2VASc≥3 (Table 1). However, these variables were not univariate predictors of primary outcome (Table 2). Recurrence in the blanking period occurred in 18 patients (16.7%) and was predictive of late recurrence only in the persistent AF group (Figure 5).

**Table 1:** – Demographic and clinical variables

	Paroxysmal AF	Persistent AF	p
N	65	43	
Age (years)	55.1±13.1	62.8±10.8	0.002
Male gender	80.0%	74.4%	0.87
Enlarged LA	20.0%	69.7%	<0.001
CHA_2_DS_2_VASc≥3	20.0%	37.0%	0.08

*AF: atrial fibrillation; LA: Left atrium.*

**Table 2:** – Variables and respective risk ratios for AF recurrence within 1 year (Univariate Cox proportional model)

	HR	IC 95%	p
Age (years)	1.02	[0.99–1.06]	0.10
Male gender	1.04	[0.35–3.06]	0.95
Enlarged LA	1.94	[0.84–4.48]	0.08
CHA_2_DS_2_VASc≥3	1.80	[0.77–4.19]	0.13
Tipo de FA	3.48	[1.41–8.59]	0.007
Recorrência BP	3.37	[1.41–8.12]	0.007

*AF: atrial fibrillation; LA: left atrium; BP: blanking period.*

**Figure 5:** – Atrial fibrillation-free survival comparing patients with or without recurrence in the blanking period by the Kaplan-Meyer curve. Parox: paroxysmal AF. Pers: persistent AF. RBP: recurrence in the blanking period.

Among the eight cases of early recurrence in the paroxysmal group, none presented late recurrence, whereas in the 10 cases of the persistent group, eight presented late recurrence.

Minor complications were observed in five patients (4.6%). One case of pericardial effusion, two cases of transient phrenic nerve palsy, with recovery in less than 15 minutes, one case of persistent phrenic nerve palsy (PNP) after hospital discharge and one case of conservatively treated femoral artery pseudoaneurysm. No major bleeding, stroke or death were observed during or after the procedure.

## Discussion

AF is the most frequent sustained arrhythmia in the general population, and regardless of the type of energy or technique used, complete isolation of the pulmonary veins is the main target for AF ablation.^2-10^ Initially, this strategy was indicated only in paroxysmal AF. However, subsequent studies demonstrated that this strategy was not inferior to more complex strategies in persistent AF.^4-9^

The Fire and Ice Study was the first large multicenter randomized trial comparing the results of cryoballoon and radiofrequency energy for ablation of paroxysmal AF and demonstrated its non-inferiority, both regarding efficacy and safety.^10,11^ Analysis of the secondary objectives of the study demonstrated benefits of the balloon considering hospitalization rates, need for cardioversion and reintervention.^11^ These benefits were confirmed by Mörtsell et al.,^13^ who recently published the efficacy and safety results of the procedure based on the ESC-EHRA and Swedish registries.

Persistent AF has a more complex substrate and the success rate with PVI is more limited.^2,14^ In order to reduce the rate of recurrence, more extensive strategies were adopted, such as additional lines and ablation of fractioned atrial electrograms.^2^ However, the additional benefit of these extensive ablations, according to recent comparative studies, remains controversial.^6,9^ Thus, according to international guidelines, PVI is still the final target of AF ablation and techniques that cover more extensive areas of ablation have not been recommended in a first intervention.^2^

Although radiofrequency ablation is considered the gold standard for persistent AF, studies with cryoenergy balloon have shown satisfactory clinical results.^13,15^

The recently published CRYO4PERSISTENT study evaluated not only the recurrence of AF, but also the presence of symptoms after PVI with cryoballoon; it demonstrated significant improvement in post-ablation quality of life.^15^ These findings were also confirmed in Mörtsell’s study,^13^ which reported less symptoms and antiarrhythmic drugs in the group that underwent cryoballoon ablation.

In our study, we reported the first experience of a Brazilian center that performed PVI using cryoballoon as the initial approach for non-pharmacological treatment of atrial fibrillation in a large number of patients. After one year, the event-free rate was 89.2% for the paroxysmal AF group and 67.4% for the persistent AF group. In the recently presented CIRCA-DOSE trial, which had its recurrence evaluated by implantable monitors, the recurrence rate was around 64%. However, symptom-free rate was close to 80%.^11-15^ In our study, the mean time to EC was 96.5±29.3 minutes and the fluoroscopy time, 29.5±11.1 minutes, which is close to the durations reported in various studies.^12,13^

Regarding the safety profile, we present a complication index of 4.6%, which is considered quite satisfactory, at levels similar to those reported in the literature.^10,12^ The most frequent complication was phrenic nerve palsy, which occurred in 3 of our patients. In two cases, paralysis was transient, reverted still in the procedure room. In one patient, paralysis was persistent, and the patient was referred to physical therapy. We present only one case of vascular complication, and we believe that the low rate of this complication is due to the fact that all punctures have been guided by ultrasound.

An important fact to be discussed is that the main destructive mechanism of cryoablation is cell lysis by intra and extracellular ice formation, causing an osmotic imbalance, leading to cell membrane rupture and damage to cell structures and, ultimately, to cell death due to coagulation necrosis and apoptosis. This leads to a weaker inflammatory response than radiofrequency (RF) ablation and, consequently, less edema,^16^ which is one of the factors identified as responsible for vein reconnection. Another important fact to be mentioned regarding procedure safety is that cryotherapy does not denature proteins and thus preserve collagen and elastin in the connective tissue and consequently preserves the extracellular matrix, which reduces the risk of thrombus formation, vein stenosis and esophagus lesion.^17^ In our group, there were no cases of esophageal fistula, clinical or laboratory evidence of pulmonary vein stenosis or death.

Our initial experience is similar to other published studies^11-16^ and confirms that results obtained with cryoablation are reproducible and less operator-dependent than those of radiofrequency ablation,^11-16^ therefore requiring a shorter learning time.

## Limitations

The main limitation of this study is that it is an observational study performed in a single center, without a control group and, therefore, there may be patient selection bias. In addition, it is an initial follow-up of a technology that is still being introduced in our country and, therefore, is not available for large-scale use, which made it difficult to include more patients.

Future randomized longer-follow-up longer-term studies should be performed to confirm our results.

## Conclusion

Cryoablation for electrical isolation of the pulmonary veins proved to be a safe effective method with an acceptable complication index and very satisfactory results. Our results are consistent with other studies that suggest that the technology can be used as the initial technique even in cases of persistent AF.
