# Supplementary material for: Errata
Source: Arq Bras Cardiol. 2020 Aug 19;115(2):302. [Article in Portuguese] doi: 10.36660/abc.20200807 (PMC8384271; doi:10.36660/abc.20200807)
Supplement: Translation

# Translation: Erratum

In the Original Article “Experience in a Brazilian Center with Crioablation for Electric Isolation of the Pulmonary Veins in Paroxysmal and Persistent Atrial Fibrillation – Preliminary Results in Brazil” with DOI number: https://doi.org/10.36660/abc.20200320, published in the periodical Arquivos Brasileiros de Cardiologia, correct the word in the English title Crioablation para Cryoablation. Correct the name of the author Eduardo Barbosa for Eduardo C. Barbosa. Change the DOI number to DOI: https://doi.org/10.36660/abc.20190307.
